# Survival and safety analysis of COVID‐19 vaccine in Chinese patients with non‐small cell lung cancer

**DOI:** 10.1002/cam4.7032

**Published:** 2024-04-23

**Authors:** Wei Xu, Jing Zhao, Fang Luan, Zhizhao Zhang, Lei Liu, Hui Zhao, Bin Feng, Guobin Fu

**Affiliations:** ^1^ Department of Medical Oncology Shandong Provincial Hospital Affiliated to Shandong First Medical University Jinan Shandong China; ^2^ Department of Medical Oncology, Shandong Provincial Hospital Cheeloo College of Medicine, Shandong University Jinan Shandong China; ^3^ Department of Medical Oncology The Third Affiliated Hospital of Shandong First Medical University Jinan Shandong China

**Keywords:** COVID‐19 vaccine safety, non‐small cell lung cancer, safety, severe acute respiratory syndrome coronavirus 2, survival analysis

## Abstract

**Background:**

Severe acute respiratory syndrome coronavirus 2 disease (COVID‐19) has caused a worldwide challenging and threatening pandemic. We aimed to assess the safety and efficacy of the COVID‐19 vaccines in Non‐Small Cell Lung Cancer (NSCLC) patients.

**Methods:**

Patient self‐reported adverse events related to vaccines were recorded by follow‐up through a uniform questionnaire. Survival analysis was performed by Kaplan–Meier method. A multivariate analysis was performed by the Cox proportional hazard regression model to determine the effect of each variable on the survival of lung cancer patients.

**Results:**

A total of 860 patients with NSCLC on treatment were enrolled. Mean age was 57 years in patients with early stage group and 62 years in advanced stage group. The vaccination rate was 71.11% for early‐stage patients and 19.48% for advanced‐stage patients; most of them (86.5%) received the COVID‐19 inactivated virus (Vero cell) vaccine (Coronavac; Sinovac). The most common systemic adverse reaction was weakness. The main reason for vaccine refusal in those unvaccinated patients was concern about the safety of vaccination in the presence of a tumor and undergoing treatment (56.9% and 53.4%). The 1‐year disease‐free survival (DFS) rate was 100% for vaccinated and 97.4% for unvaccinated early‐stage patients. Then we compared the progression‐free survival (PFS) of vaccinated (median PFS 9.0 months) and unvaccinated (median PFS 7.0 months) advanced stage patients (*p* = 0.815). Advanced NSCLC patients continued to be divided into groups receiving radio‐chemotherapy, immunotherapy, and targeted therapy, with no statistical difference in PFS between the groups (*p* > 0.05). The median overall survival (OS) of vaccinated patients was 20.5 months, and that of unvaccinated patients was 19.0 months (*p* = 0.478) in advanced NSCLC patients.

**Conclusions:**

COVID‐19 vaccination is safe for Chinese NSCLC patients actively receiving different antitumor treatments without increasing the incidence of adverse reactions, and vaccination does not affect cancer patient survival.

## 1 INTRODUCTION

The SARS‐CoV‐2 virus, which emerged in 2019 and spread quickly around the world, was named Coronavirus Disease (COVID‐19) by the WHO.^1^ The risk of severe COVID‐19 and death is much higher in cancer patients.^2^, ^3^, ^4^, ^5^, ^6^, ^7^ Almost all reports indicate that active malignancy increases risk, and risk factor like advanced age or comorbidities are also relevant to the general population.^8^, ^9^ Cancer patients progress more severely with COVID‐19 due to the natural course of the disease and the oncological treatments they receive.^10^, ^11^ In addition, the COVID‐19 pandemic has also had a significant impact on surgical care, resulting in significant delays in treatment and increased tumor size in patients with resectable early‐stage NSCLC.^12^, ^13^, ^14^ Luckily, potentially detrimental impacts can be avoided by using proactive preservative strategies and surgeon advocacy.^15^, ^16^ As a result, clinicians are challenged to manage these patients due to increased risk and need to adapt management strategies in a timely manner based on the realities of healthcare resources in each region.

It is vital for patients with cancer to prevent infection and subsequent severe COVID‐19, and vaccination is the most effective way to accomplish this. Many countries have conducted studies in order to develop a COVID‐19 vaccine since the outbreak began.^17^, ^18^ After complete vaccination doses, COVID‐19 vaccines contributed to a substantial reduction in severe COVID‐19 infections in large sections of the world.^19^, ^20^, ^21^ A variety of COVID‐19 vaccines are currently available worldwide. In China, the main types of vaccines currently are adenoviral‐vectored vaccines, inactivated vaccines, and recombinant protein‐based subunit vaccines, excluding mRNA vaccines.

Since COVID‐19 can cause severe illness in patients with cancer, most countries prioritize vaccination for them.^22^ It should be noted, however, that cancer patients were excluded from these pivotal clinical trials. Therefore, important questions concerning the efficacy and safety of currently available vaccines as well as the durability of vaccine responses remain for this population.^23^ Although some research has been initiated on this question,^24^ the efficacy and safety of vaccines administered in China are incompatible with many other countries and uncertain, so our research is needed. The fear of side effects of the COVID‐19 vaccine and the effect of anti‐cancer treatment have led to the current rate of COVID‐19 vaccine for malignant tumors in China being even lower than that of the elderly.^25^

Lung cancer remains the leading cause of cancer‐related lethality worldwide, with the five‐year survival rate is only 22%.^26^, ^27^ In this article, we examine the survival prognosis and safety of COVID‐19 vaccination in patients with non‐small cell lung cancer (NSCLC) who are undergoing active treatment. At present, statistics are not easily available on COVID‐19 vaccination in patients with NSCLC. Our study investigated the status of COVID‐19 vaccination among NSCLC patients and the influencing factors of vaccine hesitancy and provided evidence for better addressing the issues related to the special population of NSCLC patients. We also explored the incidence and types of vaccine‐related adverse reactions in the NSCLC population on active treatment. Moreover, we collected data on the prognosis of COVID‐19‐vaccinated patients with non‐small cell lung cancer and investigated whether there is a difference in the survival prognosis of patients receiving different antitumor treatment modalities to provide guidance for clinical decisions.

## 2 PATIENTS AND METHODS

### 2.1 Study design and participants

We conducted a retrospective cross‐sectional analysis to assess the safety and survival prognosis of the COVID‐19 vaccine in patients with NSCLC who had been treated. Participants in the survey were patients with any type of pathologically or cytologically confirmed non‐small cell lung cancer who received anti‐tumor treatment at our institution from June 2020 to December 2020. Telephone follow‐up or outpatient interviews were conducted between September and October 2022. Investigators collected patients' vaccination status, adverse vaccine reactions, and survival status through a uniform questionnaire. Patients who are younger than 18 with double primary tumors and SCLC were excluded.

All patients were given standardized treatment with informed consent. Anti‐tumor treatment including surgery, radio‐chemotherapy (which includes treatment with radiotherapy, chemotherapy, anti‐angiogenic drugs alone, and a combination of two or more regimens), immunotherapy (which includes immune checkpoint inhibitor therapy alone or in combination with chemotherapy), and targeted therapy (which includes treatment with molecularly targeted drugs alone or in combination with chemotherapy and immunotherapy).

### 2.2 Voluntary vaccination

Vaccination patients are vaccinated voluntarily in accordance with the regulations of the National Health Commission of the People's Republic of China, and the type of vaccine is chosen by the patient, including inactivated vaccine BBIBP‐CorV (Sinopharm) and CoronaVac (Sinovac Biotech), viral vector vaccine Convidecia (CanSino), and protein subunit vaccine Recombinant COVID‐19 Vaccine (Anhui Zhifei).

### 2.3 Clinical data collection

Relevant clinical data were retrieved from the electronic medical records of patients with cancer and included age, gender, cancer type, cancer stage (early or advanced), and diagnosis date. Among the comorbidities were autoimmune diseases, diabetes mellitus, heart disease, hypertension, and lung disease. Cancer therapies include chemotherapy, radiation, biological‐targeted therapy, immunotherapy, and surgery, either alone or in combination. A uniform standard telephone follow‐up survey was conducted using a questionnaire that included COVID‐19 vaccination status, safety, and survival rates. Based on the literature analysis and the current background of COVID‐19 vaccination, and with reference to the currently reported adverse reactions related to the COVID‐19 vaccine, the final scale was developed through expert consultation and a small sample pre‐study. Information collected through the questionnaire includes vaccination status and timing, short‐term and long‐term adverse events after vaccination, type of vaccine, patient status at follow‐up, etc.

No identifiable personal information was collected from patients in this study; the risk was considered minimal, and informed consent was waived by the Ethics Committee for Biomedical Research Involving Human Beings of Shandong Provincial Hospital (approval number: SWYX: NO. 2023‐182).

### 2.4 Safety

As to local and systemic reactions, specific yes/no questions were used to obtain adverse events. Local reactions include redness, swelling, and pain at the injection site. Systemic reactions include fatigue, fever >38°C, myalgia, chills, nausea, headache, use of palliative medicines, and vomiting.

### 2.5 Survival analysis

SPSS Statistics 23.0 was used for all analyses. Data are presented as medians and interquartile ranges for continuous covariates and percentages for categorical variables. Survival analysis was performed by Kaplan–Meier method, and the main study endpoint was progression‐free survival (PFS) and 1‐year disease‐free survival (DFS) rate. PFS was defined as the time from the prior assessment to the time the patient experienced tumor progression. DFS is defined as the time from surgical resection to local recurrence. A multivariate analysis was performed by the Cox proportional hazard regression model to determine the effect of each variable on the survival of lung cancer patients, and *p* < 0.05 indicated a statistically significant difference. These variables included age, sex, pathological diagnosis, smoking, alcohol consumption, cancer stage, and co‐morbidities.

### 2.6 Patient and public involvement

Patients or their families participated in the follow‐up survey in our study.

## 3 RESULTS

### 3.1 Patients inclusion

**FIGURE 1:**
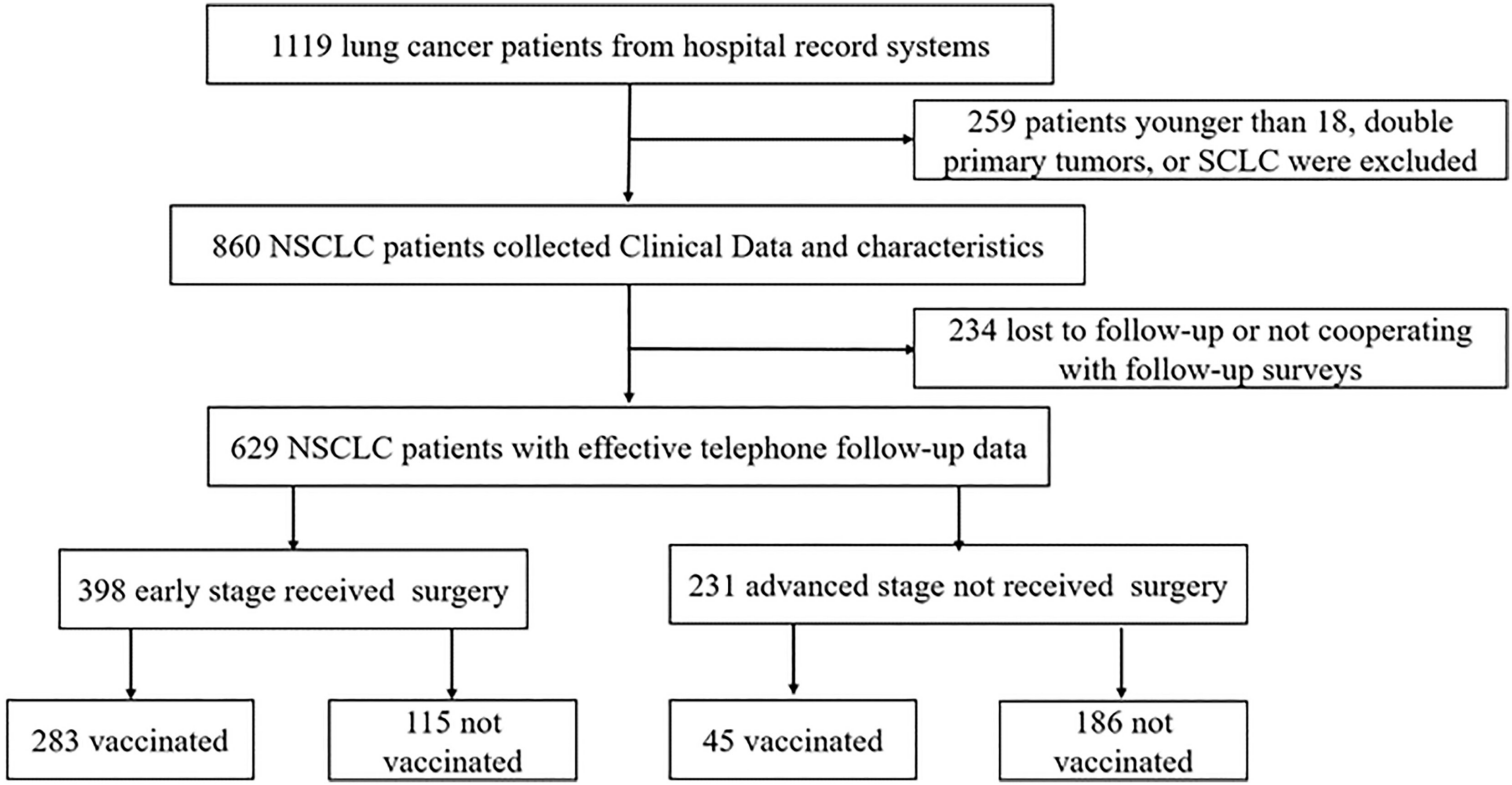
Participant flow diagram.

Between June 1 and December 30, 2020, 577 NSCLC patients with metastasis or advanced TNM stage and 542 NSCLC patients with early TNM stage receiving surgery who received anti‐tumor treatment at our institution were included in this study, and 860 patients collected clinical data and characteristics, and eventually, 629 were followed up by phone and received information on vaccination and survival (Figure 1).

### 3.2 Demographic and clinical features of participants

The main subjects and tumor characteristics of the 860 NSCLC patients are summarized in Table 1. The mean age was 57 years in patients with early stage (I or II) received surgery; 313 (58.6%) were female; and 62 years in patients with advanced stage (III or IV), 113 (34.7%) were female. Of the early stage patients, 180 (33.7%) had at least one coexisting condition in addition to cancer, and 124 (38.0%) were advanced stage patients. The most frequent medical comorbidity was hypertension (68.3% and 67.7%), followed by diabetes (30.1% and 20.2%) and cardiac disease (11.1% and 16.1%). Lung disease was more frequent in patients with advanced stages (13.0%) than in patients with early stages (5.0%). The most frequent pathological types were adenocarcinoma in both early‐stage patients receiving surgery (97.8%) and metastasis or advanced‐stage patients (75.8%), with the proportion of squamous lung cancer differs between the two population groups (2.1% and 21.5%). At the time of enrollment, 523 (97.9%) patients with early stage were post‐surgery status without adapting treatment. In patients with advanced stage, 176 (54.0%) received chemotherapy, 59 (18.1%) received targeted therapy, and 15 (4.6%) received immunotherapy. In addition, one patient has been taking Chinese herbal medicine for anti‐tumor treatment.

**TABLE 1:** Demographic and clinical characteristics of patients with early stage receiving surgery and patients with advanced stage.

	Patients with early stage received surgery (*n* = 534)	Patients with advanced stage (*n* = 326)	*p*
Age mean ± SD	57 ± 10.442	62 ± 10.183	<0.001
Gender, *n* (%)			<0.001
Male	221(41.4%)	213(65.3%)	
Female	313(58.6%)	113(34.7%)	
Pathological type, *n* (%)			<0.001
Adenocarcinoma	522(97.8%)	247(75.8%)	
SCC	11(2.1%)	70(21.5%)	
PSP	1(0.2%)	0(0%)	
LCLC	0(0%)	3(0.9%)	
Unknown	0(0%)	6(1.8%)	
Smoking status, *n* (%)			<0.001
Never	409(76.6%)	159(48.9%)	
Quit smoking	90(16.9%)	91(27.9%)	
Smoking	35(6.6%)	75(23.0%)	
Drinking state, *n* (%)			<0.001
Never	423(79.2%)	190(58.3%)	
Quit drinking	48(9.0%)	85(26.1%)	
Drinking	63(11.8%)	50(15.3%)	
Comorbidities, *n* (%)	180 (33.7%)	124 (38.0)	0.113
Diabetes	55(30.1%)	25(20.2%)	
Hypertension	123(68.3%)	84(67.7%)	
Cardiac disease	20(11.1%)	20(16.1%)	
Lung disease	9(5.0%)	16(13.0%)	
Autoimmune	6(3.3%)	2(1.6%)	
Other^a^	8(4.4%)	5(4.0%)	
Tumor stage, *n* (%)			
0	2(0.4%)	0(0%)	<0.001
I	514(96.33%)	0(0%)	
II	18(3.4%)	0(0%)	
III	0(0%)	71(21.8%)	
IV	0(0%)	255(78.2%)	
Cancer treatment, *n* (%)			
Surgery	523(97.9%)	2(0.6%)	<0.001
Chemotherapy	6(1.1%)	176(54.0%)	
Targeted therapy	9(2.7%)	59(18.1%)	
Immunotherapy	0(0%)	15(4.6%)	
Radiotherapy	0(0%)	11(3.4%)	
Unknown or others	1(0.2%)	63(19.3%)	

Abbreviations: LCLC, large cell lung cancer; PSP, pulmonary sclerosing pneumocytoma; SCC, squamous cell carcinoma; SD, standard deviation.

^a^ Pulmonary tuberculosis; hyperlipidemia; cerebral infarction; hepatitis A; hepatitis B; hepatitis C; thyroid carcinoma; chronic bronchitis; nephritis; supraventricular tachycardia; vertigo.

### 3.3 Vaccination status and safety

Since China has been adhering to a zero COVID strategy for nearly 3 years until November 2022, it has effectively protected the population from COVID‐19 infection. During our follow‐up, we did not encounter any patients who were infected with COVID‐19. Of the 629 early and advanced stage patients who were followed up, a total of 328 were vaccinated, and all of them had received at least two doses of the vaccine. Among enrolled patients, the vaccination rate was 71.1% for early‐stage patients and 19.5% for advanced‐stage patients. Vaccination‐related information is shown in Table 2. The vast majority of patients receive inactivated vaccines (86.5%). A small number of patients received adenovirus (2.1%) and recombinant protein vaccines (2.3%), and 35 patients (9.1%) were not sure which vaccine they had received at follow‐up.

**TABLE 2:** Baseline characteristics of patients with non‐small cell lung cancer who received COVID‐19 vaccine.

	*n*	%
Total	328	100
Type of vaccine		
Inactivated vaccine	287	87.5
Adenovirus vaccine	3	0.9
Recombinant protein vaccine	3	0.9
Unknown	35	10.7
Adverse reaction of the first injection	28	8.6
Local reaction^a^	15	4.6
Symptoms of URI^b^	2	0.6
Weakness	4	1.2
Myalgia	1	0.3
Rash	1	0.3
Anosmia	1	0.3
Palpitation	0	0
Allergy	0	0
Unknown	4	1.2
Adverse reaction of the second injection	32	9.8
Local reaction^a^	17	5.2
Symptoms of URI^b^	2	0.6
Weakness	2	0.6
Myalgia	2	0.6
Rash	1	0.3
Anosmia	0	0
Palpitation	1	0.3
Allergy	1	0.3
Unknown	6	1.8
Treatment plan during vaccination		
Not treated	309	94.2
Chemotherapy	6	1.8
Immunotherapy	1	0.3
Targeted therapy	9	2.7
Traditional Chinese medicine	1	0.3
Unknown	2	0.6

Abbreviation: URI, upper respiratory (tract) infection.

^a^ Redness, swelling, pain, and itching at the injection site.

^b^ Runny nose, fever, and sneezing.

**FIGURE 2:**
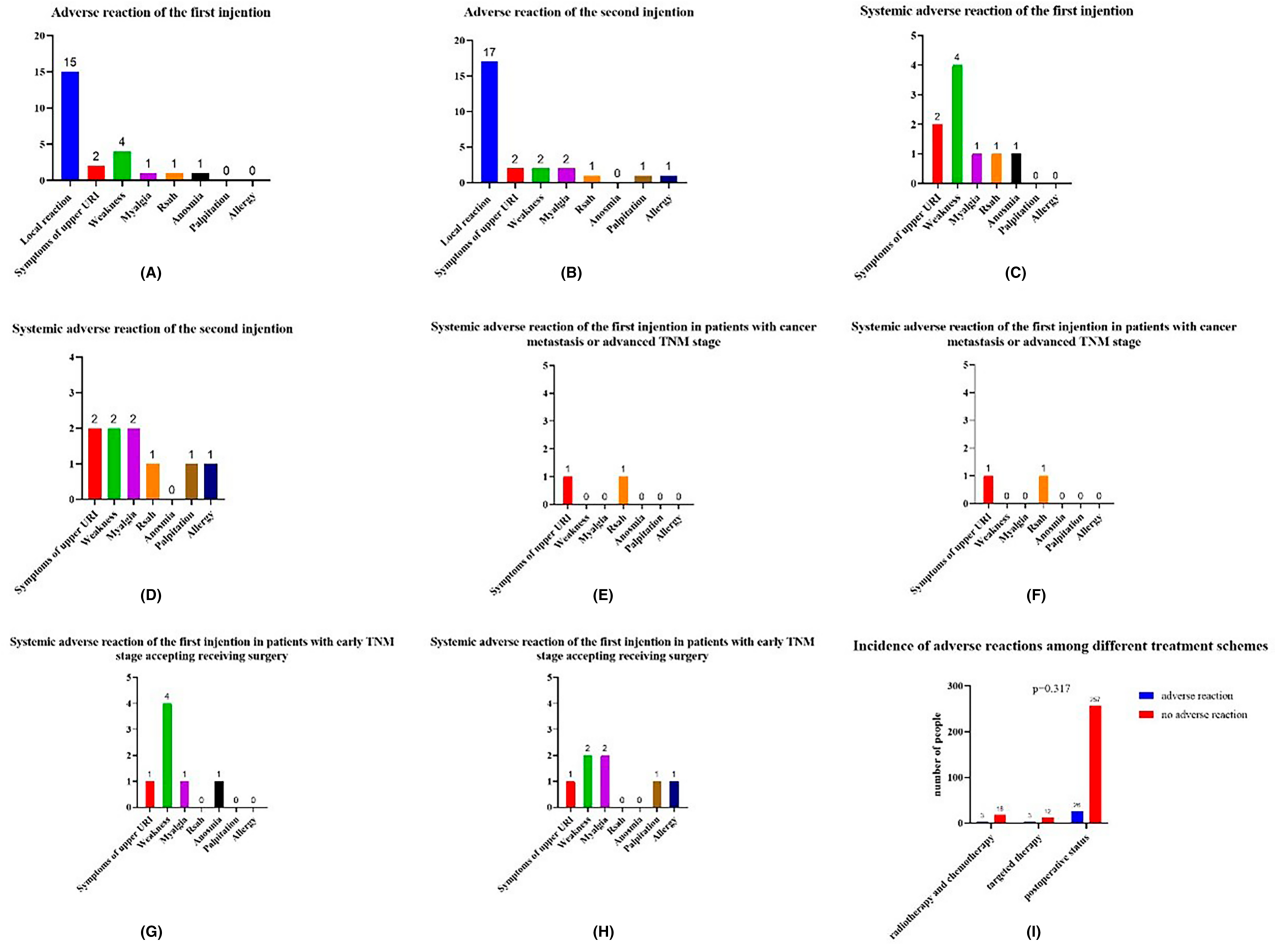
Adverse events observed after the first and second dose of vaccine. URI: upper respiratory (tract) infection. Symptoms of URI: runny nose, fever, sneezing. (A) Adverse events observed after the first dose of COVID‐19 vaccine. (B) Adverse events observed after the second dose of COVID‐19 vaccine. (C) Systemic adverse events observed after the first dose of COVID‐19 vaccine. (D) Systemic adverse events observed after the second dose of COVID‐19 vaccine. (E) Systemic adverse events observed after the first dose of COVID‐19 vaccine in patients with advanced stage. (F) Systemic adverse events observed after the second dose of COVID‐19 vaccine in patients with advanced stage. (G) Systemic adverse events observed after the first dose of COVID‐19 vaccine in patients with early stage accepted surgery. (H) Systemic adverse events observed after the second dose of COVID‐19 vaccine in patients with earl stage accepted surgery. (I) Incidence of adverse reactions among different treatment schemes.

The most common adverse reaction after both the first and second vaccination was local reaction (redness and pain at the injection site), with an incidence of 3.9% and 4.4%, respectively (Figure 2). The following systemic adverse reaction after the first dose vaccination were: weakness (1%), symptoms of upper respiratory (tract) infection (0.5%), myalgia (0.3%), rash (0.3%), and anosmia (0.3%). And the following adverse reactions after the second dose of vaccination were symptoms of upper respiratory (tract) infection (0.5%), weakness (0.5%), myalgia (0.5%), rash (0.3%), palpitation (0.3%), and allergy (0.3%). The treatment plan received at the time of vaccination was clinical watching without therapy, radio‐chemotherapy, immunotherapy, and targeted therapy. And the incidence of adverse reactions among different treatment schemes is shown in Figure 2 (*p* = 0.317).

### 3.4 Acceptance of COVID‐19 vaccines

There were 123 unvaccinated patients in the early stage and 178 unvaccinated patients in the late stage among all patients who received telephone follow‐up. We collected the reasons for vaccine refusal in these unvaccinated patients in Table 3. In both groups, more than half of the patients were concerned about the safety of vaccination in the presence of a tumor and undergoing treatment (56.9% and 53.4%). The next reason was that their doctors did not recommend vaccination (30.9% and 25.8%). In addition, there is a small group of people who do not think vaccination is necessary (6.5% and 2.2%). The remaining patients were not given a clear reason at the time of follow‐up.

**TABLE 3:** Reasons for not being vaccinated of patients with early stage and patients with advanced stage.

	Patients with early stage receiving surgery (*n* = 123)	Patients with advanced stage (*n* = 178)
Worried about the insecurity of vaccination, *n* (%)	70 (56.9%)	95 (53.4%)
Doctors do not recommend vaccination, *n* (%)	38 (30.9%)	46 (25.8%)
Unnecessary vaccination, *n* (%)	8 (6.5%)	4 (2.2%)
Other	7 (5.7%)	33 (18.5%)

### 3.5 Survival analysis of non‐small cell lung cancer patients receiving different anti‐tumor treatment regimens

**FIGURE 3:**
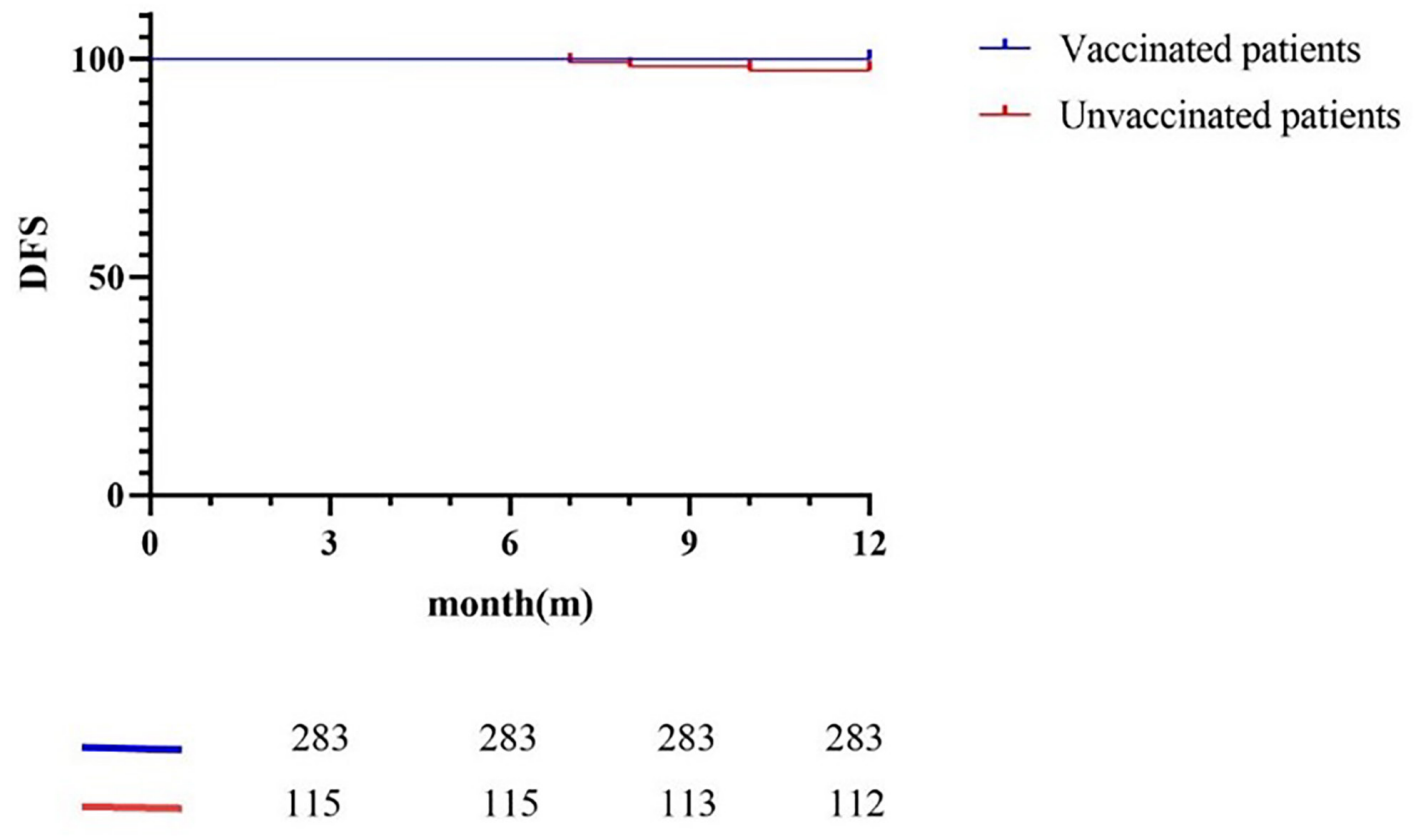
Survival curve of patients with early TNM stage accepting surgery. Two hundred and eighty‐three patients who underwent early‐stage surgery were vaccinated. These patients have no disease progression, the annual DFS rate was 100%, and 115 patients were not vaccinated. As of the observation time of 1 year, three patients had made progress, and they had made progress in the 7th, 8th, and 10th months after surgery, respectively. The annual DFS rate was 97.4%.

In the group of patients who received surgery at an early stage, 283 were vaccinated and had not progressed as of 1 year of observation time, with a 1‐year DFS rate of 100%. One hundred and fifteen were not vaccinated and 3 progressed as of 1 year of observation time, with a 1‐year DFS rate of 97.4% (*p* = 0.0064; Figure 3).

**FIGURE 4:**
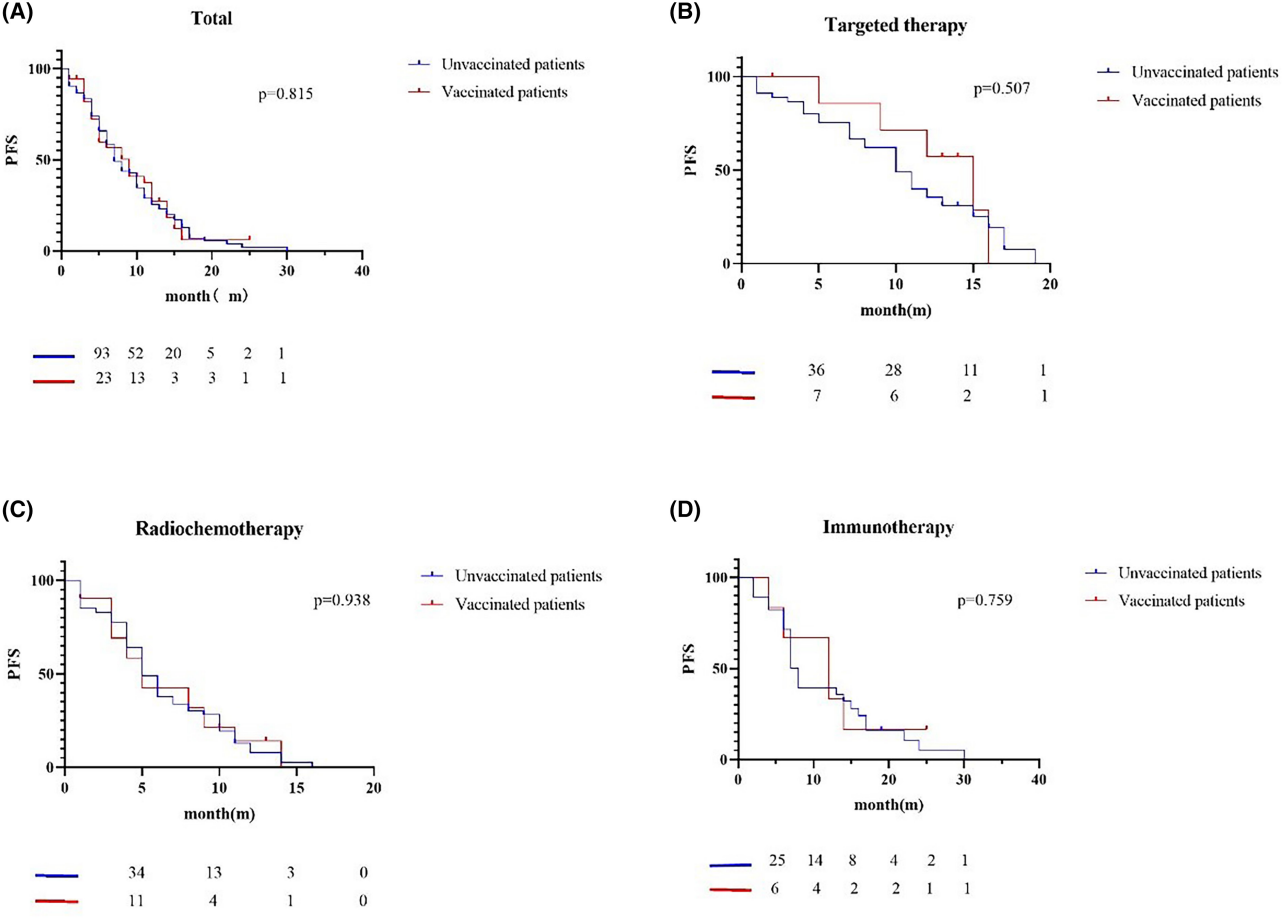
Survival curve of vaccinated and unvaccinated patients with advanced stage receiving different treatment schemes. (A) Shows the survival curve of all vaccinated and unvaccinated patients with cancer metastasis or advanced TNM stage. The median PFS of unvaccinated patients is 7.0 months, and the median PFS of vaccinated patients is 9.0 months. (B) Shows the survival curve of vaccinated and unvaccinated patients receiving targeted treatment. The median PFS of unvaccinated patients is 15.0 months, and the median PFS of vaccinated patients is 10.0 months. (C) Shows the survival curve of vaccinated and unvaccinated patients receiving radiotherapy and chemotherapy. The median PFS of unvaccinated patients is 5.0 months, and the median PFS of vaccinated patients is 5.0 months. (D) Shows the survival curve of vaccinated and unvaccinated patients receiving immunotherapy. The median PFS of unvaccinated patients is 7.5 months, and the median PFS of vaccinated patients is 12.0 months. *p* Values are greater than 0.05, and there is no statistical difference.

**FIGURE 5:**
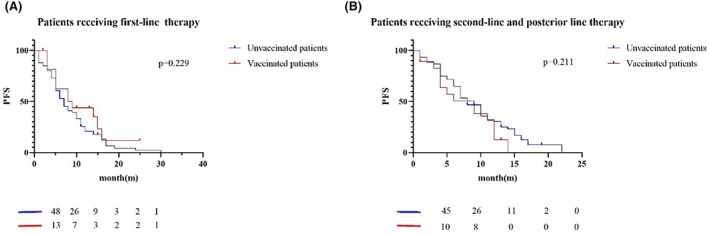
The survival curve of vaccinated and unvaccinated patients with cancer advanced stage in different treatment lines. (A) Shows the survival curve of vaccinated and unvaccinated patients with cancer metastasis or advanced TNM stage in the first line treatment. The median PFS of vaccinated patients is 8.5 months, and the median PFS of unvaccinated patients is 7.0 months. (B) shows the survival curves of vaccinated and unvaccinated patients with cancer metastasis or advanced TNM stage treated with second‐line and later lines. The median PFS of vaccinated patients is 9.0 months, and the median PFS of unvaccinated patients is 8.0 months.

In the group of patients with advanced non‐small cell lung cancer, PFS was counted in 162 individuals; of these, 35 were vaccinated and 127 were not vaccinated. The median PFS of unvaccinated patients is 7.0 months, and the median PFS of vaccinated patients is 9.0 months (Figure 4A). There was no statistical difference in PFS between vaccinated and non‐vaccinated patients (*p* = 0.815). In addition, we analyzed the impact of vaccination on the survival of patients with non‐small cell lung cancer who received different treatment regimens, including patients receiving radiotherapy or chemotherapy, patients receiving immunotherapy, and patients receiving targeted therapy. In the group of patients receiving radiotherapy or chemotherapy (Figure 4C), the median PFS was 5.0 months for the 53 unvaccinated patients and 5.0 months for the 21 vaccinated patients (*p* = 0.938). In contrast, median PFS was 7.5 months for the 29 unvaccinated patients and 12.0 months for the 6 vaccinated patients (*p* = 0.759) in those patients who received immunotherapy (Figure 4D). In addition, of those patients receiving targeted therapy (Figure 4B), median PFS was 15.0 months for the 45 unvaccinated patients and 10.0 months for the 8 vaccinated patients (*p* = 0.507). A total of 98 patients with cancer metastasis or advanced TNM stage died, 81 of whom were not vaccinated and 17 were vaccinated. We further analyzed the effect of vaccination on patients receiving first‐line and post‐line (second and post) antitumor therapy. In the group of 84 patients receiving first‐line regimens (Figure 5A), 67 were unvaccinated with median PFS 7.0 months, and 17 were vaccinated with median PFS 8.5 months (*p* = 0.229). In the group of 78 patients receiving post‐line regimens (Figure 5B), 60 were unvaccinated with median PFS 8.0 months, and 18 were vaccinated with median PFS 9.0 months. There was no statistical difference in PFS between the two groups (*p* = 0.211).

**FIGURE 6:**
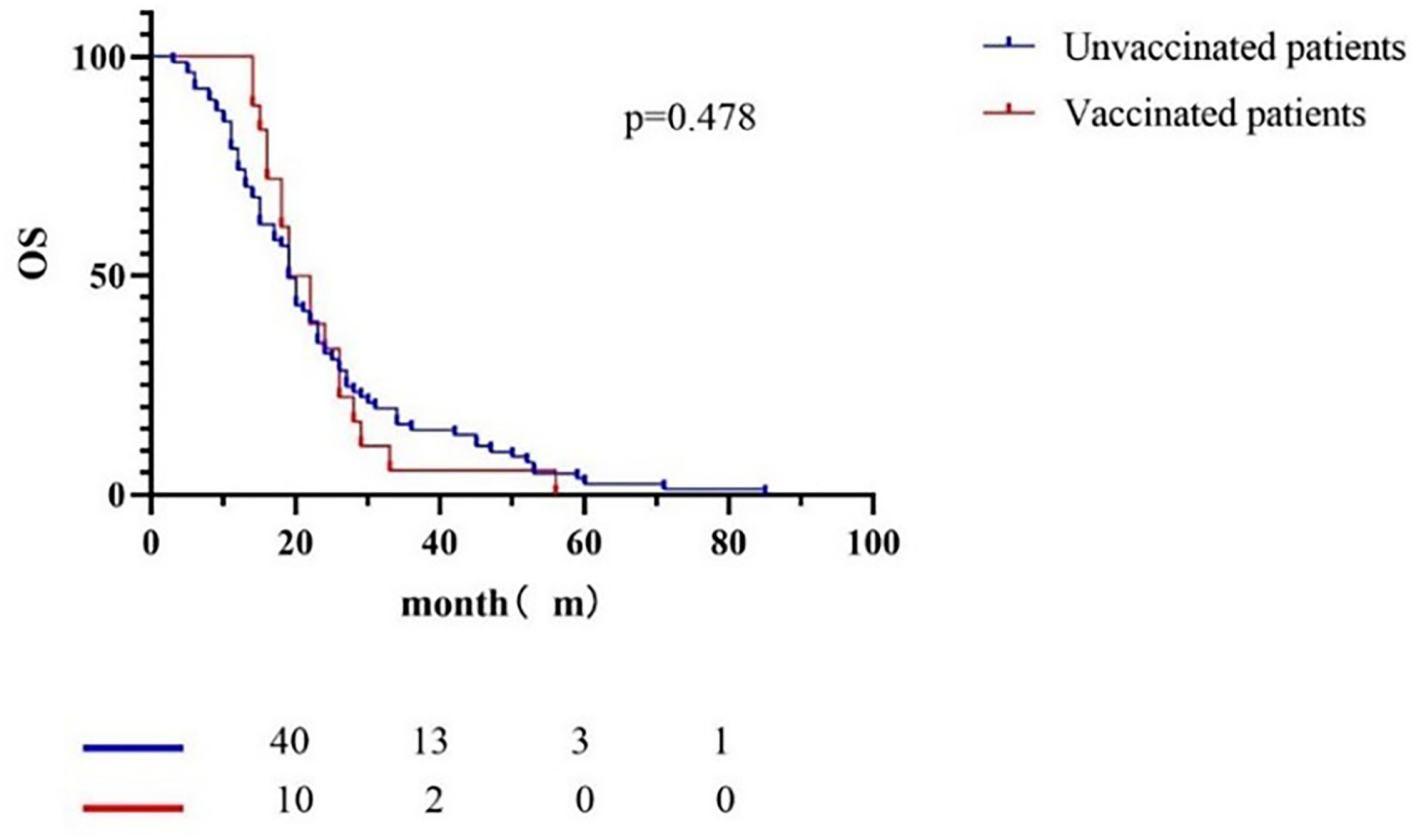
Survival curve of dead patients with advanced stage. A total of 98 patients with cancer metastasis or advanced TNM stage died, 81 of whom were not vaccinated and 17 were vaccinated. The median OS of vaccinated patients was 20.5 months, and that of unvaccinated patients was 19.0 months. The *p* value is 0.478. There was no statistical difference in OS between vaccinated patients and unvaccinated patients.

The median overall survival (OS) (Figure 6) of vaccinated patients was 20.5 months, and that of unvaccinated patients was 19.0 months (*p* = 0.478). There was no statistical difference in OS between vaccinated patients and unvaccinated patients.

## 4 DISCUSSION

The immune function of cancer patients is affected by many factors.^28^ Aggressive cancer treatments may affect immune function in cancer patients, potentially increasing the frequency and/or severity of side effects as well as reducing the effectiveness of cancer treatment. This concern has led some physicians and patients to discontinue cancer treatment or abandon vaccination at the time of COVID‐19 vaccination. Despite studies showing COVID‐19 vaccination is safe for cancer patients^28^, ^29^ and that the severity of COVID‐19 infection does not increase with ICI treatment,^30^ to date, data on the safety and prognostic impact of COVID‐19 vaccination and cancer treatment are scarce.

Our study statistically confirms that SARS‐CoV‐2 vaccination does not increase side effects in actively treated Chinese NSCLC patients. This study reports for the first time the real‐world status of vaccination in patients with non‐small cell lung cancer receiving different anti‐tumor treatment regimens and analyzes the survival of these patients. The results showed no significant difference in PFS between vaccinated and unvaccinated patients in a population of non‐small cell lung cancer patients receiving radiotherapy, immunotherapy, or molecular targeted therapy. For those patients with early‐stage tumors who underwent surgery, there was again no significant difference in 1‐year DFS between the vaccinated and unvaccinated subgroups. In addition, the incidence of adverse reactions in vaccinated patients with advanced non‐small cell lung cancer was also similar to that in postoperative patients with early‐stage non‐small cell lung cancer.

According to data released by the National Health Commission of the People's Republic of China, the vaccination rate for the first dose of vaccine for people over 60 years of age in China has exceeded 90%; however, vaccination guidelines recommend that vaccination be withheld for serious chronic diseases in their acute phase, such as cancer patients undergoing chemotherapy. The results of this study showed that the vaccination rate was higher in patients with early‐stage non‐small cell lung cancer who underwent surgery (71.1%) compared to patients with advanced non‐small cell lung cancer (19.48%), but still lower than in the elderly population over 60 years of age. This suggests that vaccination of patients with non‐small cell lung cancer is influenced by tumorigenesis, especially in patients with advanced disease. We further analyzed the reasons why patients were not vaccinated in our groups. And the main reason is that patients are concerned about the safety of COVID‐19 vaccination while receiving anti‐tumor therapy, and they also worry that vaccination will affect the effectiveness of anti‐tumor therapy, that's exactly what we're working on. This is similar to the results of a study investigating the attitudes and factors of breast cancer patients who were hesitant to receive the COVID‐19 vaccine,^31^ in which the most important reason among those hesitant to receive the COVID‐19 vaccine was concern about the safety of the vaccine in the oncology population. In addition, there are still some patients who are not aware of the need for COVID‐19 vaccination. For those cancer patients who are eligible for vaccination, we need to increase awareness of the role of vaccination, and the patient's primary care physician should give appropriate advice.

Multiple studies report safety of SARS‐CoV‐2 mRNA vaccine in cancer patients.^32^, ^33^, ^34^ In our study population, the vast majority were vaccinated with inactivated vaccines. A study evaluated the short‐term safety of BBIBP‐CorV in patients receiving ICIs.^34^ In such cases, the COVID‐19 vaccine BBIBP‐CorV does not increase serious anti‐PD‐1‐related adverse events or reduce the clinical efficacy of camrelizumab (a PD‐1 antibody approved in China) in cancer patients. In the present study, there was no increase in the incidence of adverse events over a relatively long period of time compared to historical data, which is consistent with the above information. To our knowledge, this study is the first to analyze the effect of vaccination on the survival of patients with non‐small cell lung cancer. Because many patients are concerned that vaccination affects the efficacy of antineoplastic therapy, there is a lack of relevant evidence to deny this idea. Our results show that COVID‐19 vaccination has no significant effect on survival in patients with non‐small cell lung cancer receiving first‐line or post‐line radiotherapy, chemotherapy, immunotherapy, and targeted therapy. This provides some real basis for the safety of the COVID‐19 vaccine in patients with non‐small cell lung cancer.

Our study has its limitations. First, we are a retrospective study, which reduces its clinical impact. Second, this cohort did not collect laboratory results for detailed immune function analysis to investigate the effect of antitumor therapy on the efficacy of COVID‐19 vaccination, despite the fact that no cancer patients in the vaccination group were infected with SARS‐CoV‐2. Future studies will be necessary to investigate these issues.

## AUTHOR CONTRIBUTIONS

**Wei Xu:** Conceptualization (equal); data curation (equal); investigation (equal); methodology (equal); writing – original draft (lead). **Jing Zhao:** Conceptualization (equal); formal analysis (equal); methodology (equal); project administration (equal); validation (equal); writing – original draft (supporting). **Fang Luan:** Investigation (equal); software (equal); writing – original draft (supporting). **Zhizhao Zhang:** Investigation (equal); writing – original draft (supporting). **Lei Liu:** Investigation (equal); software (supporting); writing – original draft (supporting). **Hui Zhao:** Investigation (equal); writing – original draft (supporting). **Bin Feng:** Conceptualization (equal); formal analysis (equal); methodology (equal); project administration (equal); resources (equal); supervision (equal); writing – review and editing (equal). **Guobin Fu:** Conceptualization (equal); data curation (equal); formal analysis (equal); funding acquisition (equal); methodology (equal); project administration (equal); resources (equal); supervision (equal); writing – review and editing (lead).

## FUNDING INFORMATION

This work was supported by the National Natural Science Foundation of China (81802284); Taishan Scholar Foundation of Shandong Province (tsqn202103179); 2021 Shandong Medical Association Clinical Research Fund (YXH2022ZX02176) and Beijing Xisike Clinical Oncology Research Foundation (Y‐HR2022MS‐0257).

## CONFLICT OF INTEREST STATEMENT

All authors declare that there is no conflict of interest.

## ETHICS STATEMENT

The studies collected in this paper were obtained from public repositories through legal means, and the cited studies were reviewed by the Ethics Committee for Biomedical Research Involving Human Beings of Shandong Provincial Hospital and did not violate any ethical principles (approval number: SWYX: NO.2023‐182).

## Data Availability

The data used to support the findings of this study are available from the corresponding authors upon request.
